# Practical routes to preregistration: a guide to enhanced transparency and rigour in neuropsychological research

**DOI:** 10.1093/braincomms/fcaf162

**Published:** 2025-04-28

**Authors:** Richard J Binney, Laura J Smith, Stephanie Rossit, Nele Demeyere, Gemma Learmonth, Elena Olgiati, Ajay D Halai, Elisabeth Rounis, Jonathan Evans, Nicola M J Edelstyn, Robert D McIntosh

**Affiliations:** The British Neuropsychological Society, Open Research Group, London, UK; Department of Psychology, Bangor University, Bangor, Wales LL57 2AS, UK; The British Neuropsychological Society, Open Research Group, London, UK; Wolfson Institute of Population Health, Queen Mary University of London, London EC1M 6BQ, UK; The British Neuropsychological Society, Open Research Group, London, UK; School of Psychology, University of East Anglia, Norwich NR4 7TJ, UK; Nuffield Department of Clinical Neurosciences, Oxford University, Oxford OX3 9DU, UK; The British Neuropsychological Society, Open Research Group, London, UK; Faculty of Natural Sciences, University of Stirling, Stirling FK9 4LA, UK; The British Neuropsychological Society, Open Research Group, London, UK; Department of Brain Sciences, Imperial College London, London W12 0NN, UK; The British Neuropsychological Society, Open Research Group, London, UK; MRC Cognition and Brain Sciences Unit, University of Cambridge, Cambridge CB2 3EF, UK; The British Neuropsychological Society, Open Research Group, London, UK; Department of Brain Sciences, Imperial College London, London W12 0NN, UK; MRC Cognition and Brain Sciences Unit, University of Cambridge, Cambridge CB2 3EF, UK; School of Health and Wellbeing, University of Glasgow, Glasgow G12 8TB, UK; The British Neuropsychological Society, Open Research Group, London, UK; School of Sciences, Bath Spa University, Bath BA2 9BN, UK; The British Neuropsychological Society, Open Research Group, London, UK; School of Philosophy, Psychology and Language Sciences, University of Edinburgh, Edinburgh EH8 9JZ, UK

**Keywords:** open research, reproducibility, publication bias, clinical science, best practice

## Abstract

Preregistration is the act of formally documenting a research plan before collecting (or at least before analysing) the data. It allows those reading a final research report to know which aspects of a study were decided before sight of the data, and which were added later. This enables informed evaluation of the severity with which scientific claims have been tested. We, as the British Neuropsychological Society Open Research Group, conducted a survey to explore awareness and adoption of open research practices within our field. Neuropsychology involves the study of relatively rare or hard-to-access participants, creating practical challenges that, according to our survey, are perceived as barriers to preregistration. We survey the available routes to preregistration, and suggest that the barriers are all surmountable in one way or another. However, there is a tension, in that higher levels of bias control require greater restriction over the flexibility of preregistered studies, but such flexibility is often essential for neuropsychological research. Researchers must therefore consider which route provides the right balance of rigour and pragmatic flexibility to render a preregistered project viable for them. By mapping out the issues and potential solutions, and by signposting relevant resources and publication routes, we hope to facilitate well-reasoned decision-making and empower neuropsychologists to enhance the transparency and rigour of their research. Although we focus neuropsychology, our guidance is applicable to any field that studies hard-to-access human samples, or involves arduous or expensive means of data collection.

## Teaser

Binney *et al*. examine the benefits of preregistration in neuropsychology, and several routes to achieving the key objectives. They survey perceived barriers, highlighting tension between bias control and a need for flexibility in studying rare or hard-to-access populations. They argue these barriers are surmountable and provide solutions applicable beyond neuropsychology.

## Graphical Abstract

**Graphical Abstract:**
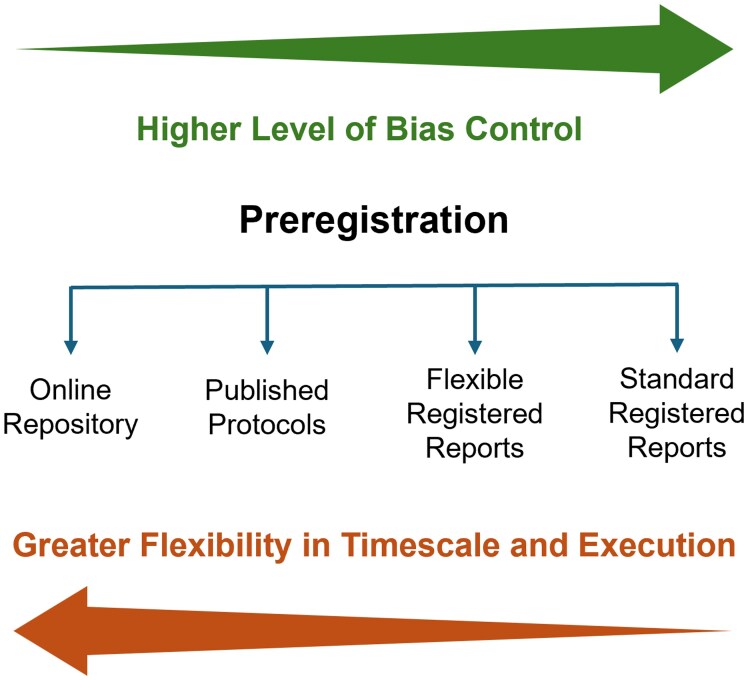

## Preregistration: what, why and how

Preregistration means documenting the details of a research plan before carrying out that study. This is more than private planning, because the preregistration document must be timestamped and persistent, so that it cannot be altered without record. The document can be shared immediately or placed under embargo for some period, but it must ultimately be open to scrutiny. This transparency confers an essential benefit over and above the advantages of careful planning and record-keeping: it allows readers to know which aspects of a study were decided before sight of the data, and which were added later. In a hypothesis-testing context, a good preregistration will clearly specify the hypotheses and the analyses that will test them. This reduces subsequent scope for undisclosed flexibility in analysis choices^1^ and for revisionist reframing of hypotheses to align with results (i.e. HARKing: hypothesising after the results are known).^2^ Effective preregistration allows readers to evaluate the severity with which hypotheses have been tested. ‘Severity’ is used here in the technical sense used by Mayo,^3,4^ which relates to the capacity of a test to falsify, and not just confirm, a prediction. Lakens^5^ argues that, while preregistration does not ensure severe tests, it crucially does allow readers to evaluate how severely a hypothesis has been tested. As the scientific value of open practices is increasingly recognized, these practices may become more rewarded by funding agencies and hiring and promotions panels, adding professional incentives for engagement.

As we discuss in more detail below, we presently see four main routes to preregistration. These entail different levels of detail of documentation, different degrees of external scrutiny, and allow more or less flexibility in study execution. These differences affect the level of bias control that can be achieved, and the time investment allocated to different stages of the process. The lightest-touch and most malleable approach is repository preregistration. Here, researchers upload a study plan to an online repository, ideally before the study begins, but certainly before the data are analysed. This creates a timestamped preregistration document that can be referenced/linked to in the final research report. A second option is to publish a study protocol as a standalone paper, creating a citable, peer-reviewed record of the plan. The third and strongest form of preregistration is the Registered Reports article type, in which the Introduction and Methods for a study are reviewed at a journal or other peer review platform before the study begins. A favourable outcome is accompanied by an *in principle* editorial commitment to publish the final empirical report regardless of how the results turn out. Finally, more flexible versions of the Registered Reports format are now emerging, which deserve consideration in their own right.

The existence of a preregistration record should not be viewed as a proxy for study quality. Preregistration documents can be poor, and poorly-designed studies can be preregistered, just as research of the highest quality can be done without preregistration. Indeed, some fundamentally valuable forms of research may not be suited for preregistration, for instance studies that are open-ended and exploratory, and more concerned with hypothesis-generation than hypothesis-testing.^6^ Even the strongest form of preregistration, Registered Reports, depends crucially upon the quality and rigour of the editorial and peer review process. Nonetheless, *all other things being equal*, preregistration increases transparency, enhancing the value of the research by allowing readers to better assess whether the results are likely to be reliable.

The purpose of this paper is to provide guidance to researchers in neuropsychology about the various routes to preregistration, and to help break down barriers to adopting this practice. It emerged from discussions amongst the executive committee of the British Neuropsychological Society, who have identified open research as a priority for the field. Our focus is on neuropsychology, but the guidance is also applicable to cognate disciplines such as behavioural neurology and neuropsychiatry, and any other field that studies hard-to-access human samples, or involves arduous or expensive means of data collection. We will discuss the pros and cons of the main routes to preregistration, after first considering the special constraints and challenges that apply to neuropsychological research. By mapping out the issues, we hope to facilitate well-reasoned decision-making, helping researchers identify practical routes to enhancing the transparency and rigour of their studies.

## Challenges for neuropsychology

Neuropsychology is the study of cognition, affect, and behaviour in people with damage, disease, or divergence of the brain or nervous system. It incorporates diverse approaches, from observational studies and experimental behavioural methods to structural and functional brain imaging and neurostimulation. The emphasis may be on basic questions about the functional organisation of the brain, or clinically-oriented with an applied focus on diagnosis, prognosis, or treatment of a neuropsychological condition. For the present discussion, the unifying feature of neuropsychological research is the study of relatively rare or hard-to-access participants. The study of such samples intensifies the challenges of research, and could reduce the appetite or ability to deal with the additional requirements of preregistration.

**Figure 1:**
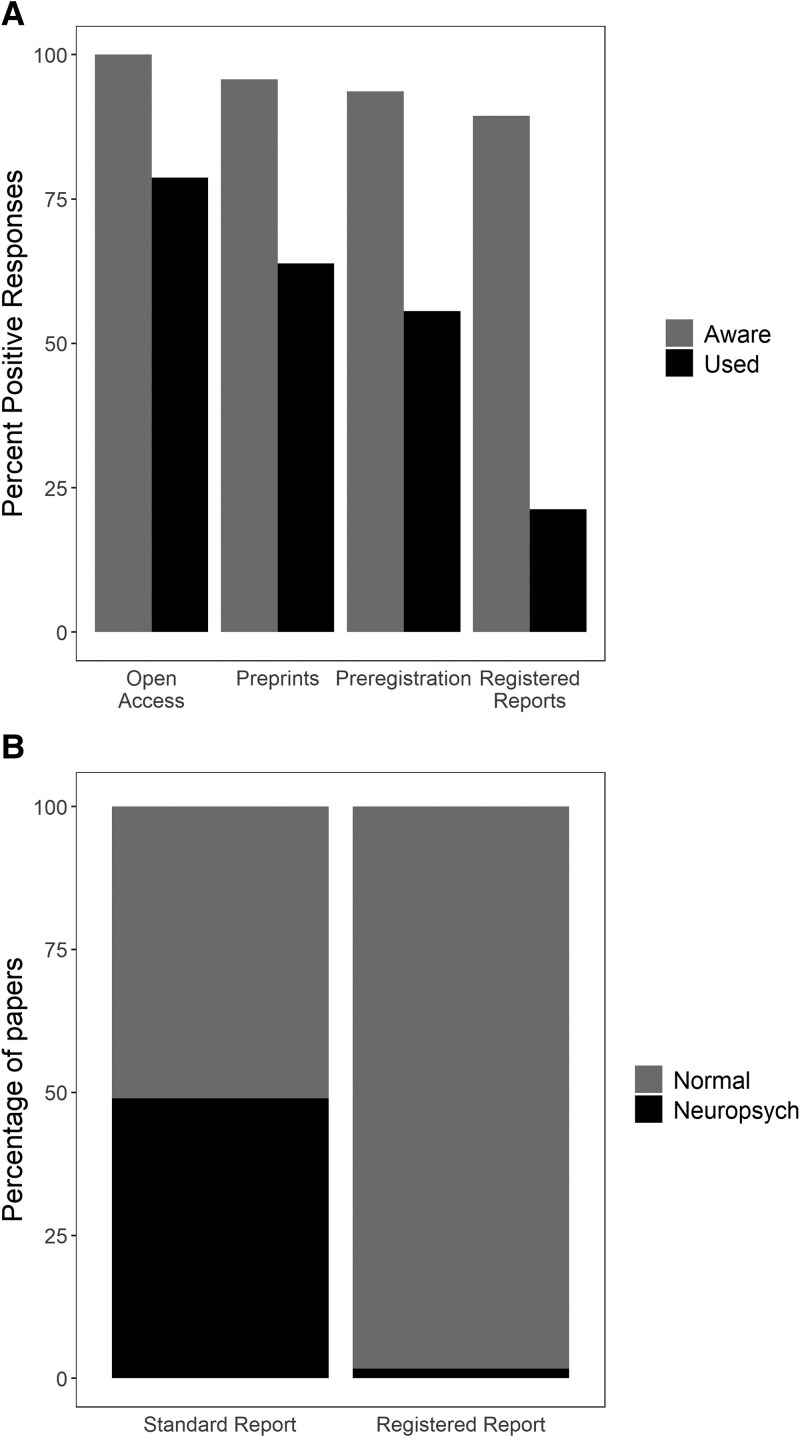
**Results from British neuropsychological society survey of research-active neuropsychologists.** (A) Percentage of respondents (*n* = 47) reporting awareness (grey) and use (black) of study preregistration, and Registered Reports, as compared with the two most well-known and used categories of open research practice (Open Access, Preprints). (B) Results from audit of Cortex editorial records for standard research articles accepted in 2022 (*n* = 145) and Registered Reports accepted in 2018–2022 (*n* = 62), showing percentage of studies using normal healthy participants only (grey) or samples of people with a neuropsychological condition (black).

To explore this issue, we recently surveyed research-active UK neuropsychologists about their awareness of and engagement with a variety of open research practices, including preregistration and Registered Reports. The survey was adapted from the Brief Open Research Survey developed by members of the UK Reproducibility Network.^7^ Respondents were almost all members of the British Neuropsychological Society and/or the British Psychological Society, Division of Neuropsychology. It is likely that members with an active interest in open research were the most willing to complete the survey, so the responses of the small sample (*n* = 47) may be biased in this regard. Figure 1A shows summary data for the two forms of study registration in the survey (preregistration, and Registered Reports) compared with two other categories of open research practice (Open Access publication, and preprints). The reported use of preregistration (>50%) is encouragingly high as benchmarked against use of Open Access (∼70%) which is increasingly a normative practice of publication. It should be noted, however, that the survey did not isolate research using neuropsychological samples, and so some of this engagement could represent research with healthy neurotypical participants (and other research approaches, such as secondary data analyses). It may also include clinical trials, in which preregistration of study plans within an online repository is common, and often mandatory (see below).

By contrast, reported engagement with Registered Reports was relatively low, and so we took an opportunity to explore this further via other means. The Registered Reports format was first established at the journal *Cortex*,^8^ which is a premiere outlet for neuropsychological research. With the help of the *Cortex* journal office, we audited all Registered Reports (*n* = 62) accepted for publication across 5 years (2018–2022), which account for ∼10% of all research articles accepted during this period. We classified each Registered Report according to whether it included a neuropsychological sample (broadly defined) or neurotypical participants only. For comparison, we made the same classification for all standard research reports accepted at *Cortex* in 2022 (*n* = 145). While nearly half of the standard research articles involved neuropsychological samples, only one of the 62 Registered Reports did^9^ (see Fig. 1B). This stark difference suggests major obstacles to the use of Registered Reports for neuropsychological research.

Around half of respondents in our survey (23/47) agreed that there are barriers to engagement with open research that apply particularly to neuropsychology. A few (3/23) raised concerns about the difficulty of preregistering single-case designs, a distinctive form of neuropsychological research that we will address in a later section. However, the most common concern (12/23) was related to the public sharing of potentially sensitive data from clinical samples. This raises the possibility that, while preregistering research does not necessarily require ‘open data’, the two practices are so strongly associated in researchers’ minds that concerns over data sharing can discourage preregistration. Meyer^10^ has written a thoughtful tutorial containing practical tips for effective and ethical sharing of data from human participants. They highlight that there is no one-size-fits-all approach to generating open data, and demonstrate several means by which the accessibility and, ergo, the value of datasets can be maximized while avoiding added risk to participants. In a later section, we expand on this to consider whether restrictions and decisions around data sharing practices affect preregistration and the level of bias control that can be achieved.

An overlapping subset of respondents (23/47) identified more general barriers to engaging with open research. The most common (9/23) was the extra time and effort associated with preregistration and open data. This concern applies across all disciplines,^7^ but bites especially hard for those with other major challenges for participant recruitment and study execution. In Table 1, we summarize various challenges that affect neuropsychological research, and highlight how they could be perceived to conflict with the requirements of preregistration. We will address these real and perceived barriers, and consider how they may be overcome, as we describe different routes to preregistration.

**Table 1:** Examples of common baseline challenges of neuropsychological research which interact with perceived requirements of preregistration

Resource	Baseline challenges for neuropsychology	Perception of preregistration
**Time and sequencing**	Studies often lengthy, difficulty recruiting participants, complexity co-ordinating clinical teams, scheduling and testing. Clinical ethical approvals can take many months, with further background checks and bureaucracy to be completed. Clinical ethical approvals are very specific, so all changes require amendments, and major changes require substantial amendments.	Additional steps inserted before study can begin. Requires existing ethical approvals, so must be serialized to ethics process. Peer-reviewed preregistrations may entail changes to methods.
**Sample size and power**	Recruitment of large participant samples may be difficult or practically impossible. Clinical ethics committees consider patient burden and resource use, and oppose ‘overpowered’ designs. Single-case statistical comparisons are inherently low in achievable power.	Requires high power, which implies large sample sizes. Prioritises ideal designs over practical considerations. Requires high power, which may be unachievable for single-case studies.
**Flexibility of study execution**	Limited control over testing schedules and environment. Data collected opportunistically, and contingent on individual participant abilities; entails variations of protocol, missing data. Recruitment mechanisms and inclusion/exclusion criteria may need to be adjusted to meet recruitment targets. Single-case approaches often reactive, designed in response to patient’s condition; cannot be defined in advance.	Preregistration requires exact adherence to plan.
**Focus and scope**	Difficulty of recruitment means data collection should be maximized, collecting as much information as feasible, for further context or possible future interest.	Preregistered studies target specific hypotheses, not suited to exploratory aims.
**Data restrictions**	Clinical data often sensitive, and there may be elevated concerns over anonymity, and possibility of identification.	Preregistered publication routes require sharing of raw data.

## Routes to preregistration

The scientific and practical implications of four main routes to preregistration are summarized in Table 2. There is a general antagonism, such that the highest levels of scientific benefit in terms of unbiased execution require greater restriction over the flexibility and time-course of the research. Lighter-touch forms of preregistration are less guaranteed to control bias, but may nonetheless be the most pragmatic choice for some studies. In general, researchers need to consider which route provides the right balance of rigour and flexibility for their particular project. To inform this decision process, we will discuss each route in more detail.

**Table 2:** Relative scientific and pragmatic benefits of alternative preregistration routes

Scientific (S) and pragmatic (P) benefits of preregistration route	Repository preregistration	Published protocols	Standard registered reports	Flexible registered reports
**Control of researcher bias (S)**	Low	Mid	High	Variable
**Control of publication bias (S)**	Low	Low	High	High
**Level of transparency (S)**	Low	Mid	High	High
**Peer-reviewed (S)**	No	Yes	Yes	Yes
**Flexibility of execution (P)**	High	High	Low	Mid
**Freedom of timecourse (P)**	High	Mid	Low	Mid
**Additional research output (P)**	No	Yes	No^a^	No*
**Guaranteed final publication (P)**	No	No	Yes	Yes

Higher levels of bias control and transparency generally require greater practical constraints.

^a^Note that the Registered Reports Stage 1 manuscript can provide an interim peer-reviewed citable object if it is archived publicly prior to completion of the study.

### Repository preregistration

The simplest form of preregistration is to post the study design to an online repository. This general system was first established for clinical trials of health interventions. The original purpose was to facilitate the discovery of ongoing trials by clinicians and prospective participants, but the potential to reduce bias in the reporting of outcomes was soon recognized as a further benefit.^11^ Indeed, in the UK, approval of a clinical trial from a National Health Service Research Ethics Committee now carries the requirement that the study protocol be formally registered in line with World Health Organisation (WHO) criteria (for content, quality, accessibility, technical capabilities, etc; www.who.int/clinical-trials-registry-platform/network/registry-criteria). These registrations are public, though an embargo period may be allowed for scientifically or commercially sensitive trials. If the data are subsequently reported, the timestamped registration adds transparency, because deviations from the original plan can be identified by any sufficiently interested reader.

Over the past decade, the general method of repository preregistration has spread beyond clinical trials to other forms of research, becoming mainstream within psychology and related fields. Prominent repositories include the Open Science Framework (OSF), and the AsPredicted platform hosted by the University of Pennsylvania. A preregistration document should contain core information about study hypotheses, measures, sampling plan, and data processing steps, including statistical tests and the conclusions they will underpin. To provide guidance and to ensure coverage of essential details, the main platforms provide authors with a preregistration template or form. The AsPredicted template includes a minimal question set, with information collected under 11 different prompts. The standard OSF template has 14 mandatory questions with multiple further fields for optional information. The responses to these questions are not peer-reviewed, although there is currently a light moderation process at the OSF to screen out violations of basic standards. Repository preregistration therefore guides researchers and encourages careful planning, but leaves them in full control, with no substantive checks on the quality or completeness of the study plans. The feature that distinguishes a preregistration document from an online lab-book is that, once uploaded to a repository, it cannot be deleted. However, this does not imply that the preregistration will always be discoverable, which depends on the public access policy. Preregistrations can be kept private for a maximum of 4 years at OSF, but can remain private indefinitely at AsPredicted. Under some circumstances, preregistrations can be ‘withdrawn’, in which case only minimal details of the original plan may be preserved. However, where the preregistration is made public, as intended, it allows readers to better evaluate the severity with which a claim has been tested.^5^

The purpose of the preregistration document is to encourage careful planning and improve transparency, not to tie the researchers’ hands in study execution. The preregistration is ‘a plan not a prison’, and if the plan needs to change as the study progresses, that is at the authors’ discretion.^12^ On some platforms it is currently possible to make a timestamped update, or to append the registration with further information (e.g. additional measures, or altered analytical choices). It is best practice to note any changes (or ‘deviations’) in the final report, along with a rationale for them. However, authors should not necessarily expect that their prospects for publication will be enhanced by the existence of a preregistration. Indeed, few if any journals require editors and reviewers to check preregistration documents, or provide resources or recognition for such thoroughness. This is true even where papers are explicitly ‘badged’ in recognition of engagement with the preregistration process.^13^ There is no guarantee that a published study with a linked preregistration has been reviewed for adherence to the preregistered study plan, nor that the rationales for any deviations have been evaluated. Nonetheless, the existence of a preregistration does signal a willingness to open up these details for scrutiny.

In sum, repository preregistration is a common requirement for clinical trials, and a relatively low-cost add-on for studies of any sort. It encourages detailed forethought, increases transparency, and represents a step towards best practice. It has the merits of efficiency and flexibility because a preregistration template can be completed relatively quickly, and updated or deviated from as necessary. For researchers in neuropsychology, it is a pragmatic route that can accommodate some of the challenges outlined earlier. However, repository preregistration is the weakest route for controlling possible sources of bias, and meta-scientific evidence has shown that the quality of repository preregistrations is very variable,^14-17^ and that biases towards positive findings may persist even with preregistration.^18^ We advise researchers to use this process as responsibly as possible (i.e. to make detailed preregistrations and document any deviations), but to be realistic about its limitations. We view it as an accessible tool for focusing attention on advance planning, but this is challenging to do well without external input.

### Published protocols

Published protocols refers to the model of publishing a study protocol as a standalone research output, prior to publication of the full study report. Like the original online registries for study plans, published protocols have developed within the extended ecosystem of clinical trials, and the majority of journals offering this option are focused on clinically-oriented research. However, the model may be applicable to basic as well as applied neuropsychological research, provided that a suitable outlet can be identified (see Table 3 for a non-exhaustive list of outlets for protocols, along with some notes on primary aspects of their process, for instance, the eligibility criteria and peer review mechanism). Below we sketch how this route can be a valuable option for our field, albeit with limitations.

**Table 3:** Possible outlets for publishing protocols in neuropsychology

Journal/Journal group	Criteria and process	Publishing model
BMC journals Inc. Geriatrics, Pilot and Feasibility Studies, Psychiatry, Psychology, Public Health, Stroke and Vascular Neurology, Trials.	Proposed/ongoing studies that have not completed participant recruitment. Fast-track process for studies with formal ethical approval or major funding.	Gold Open Access
European Stroke Journal	Randomised controlled trials and other clinical trials only. Scheduled or ongoing studies.	Subscription or Gold Open Access.
F1000 Research	Any type of design can be submitted. Protocol template provided with guidance for specific study types.	Diamond or Gold Open Access.
Frontiers Journals Inc. Cognition, Dementia, Human Neuroscience, Psychiatry, Psychology, Stroke.	Other articles relating to the study must not already be published or in review. Pilot and feasibility protocols are not accepted. Guidance for specific study types provided.	Gold Open Access
International Journal of Stroke	Major clinical trials (phase III) or major observational/epidemiological studies only. Trials should have begun but not finished recruitment. Clinical trials must be registered on WHO-approved trials registry and referenced against CONSORT statement.	Gold Open Access
Journal of Medical Internet Research (JMIR) Research Protocols	Publishes research ideas, grant proposals and study protocols in all areas of medicine. In principle acceptance for publication of subsequent results in JMIR journals if protocol adhered to. Guidance for specific study types provided.	Gold Open Access
PLOS ONE	Proposed/ongoing studies that have not completed participant recruitment or data collection. Guidance for specific study types provided.	Gold Open Access
Translational Stroke Research	Instructions of what information to include in the protocol are provided.	Subscription or Gold Open Access.

The main candidates are either clinical journals (e.g. BMJ-O), more general journals (e.g. PLOS; F1000Research), or protocol-only outlets (e.g. JMIR Research Protocols). Three stroke-specific outlets publish protocols, but options were lacking for other disease-specific journals relevant to neuropsychologists. Some publishers restrict eligible study designs, for instance to clinical trials or systematic review protocols. These various restrictions may have implications for readership, as well as the pool of likely reviewers with disciplinary expertise.

First, unlike online repository preregistration, the publication of a protocol involves peer-review. This creates a formal opportunity to receive interim feedback on the study design and potential implications. The review process may reveal errors, ambiguities, or areas of contention, and offers the opportunity to enhance the rigour and framing of the work. Editors at participating journals are likely to have a good appreciation of the challenges inherent in clinical trials, including the fact that funding agreements and the conditions of ethical approval can limit design flexibility. On the other hand, there may still be scope for the fine-tuning of methods, and in particular of analysis plans, and the focus of the peer-review may often lie here. Some journals offer a streamlined, lighter-touch review track (e.g. kept within the editorial board) for protocols that have already passed through external peer review as part of a funding or ethics process. Some outlets will consider publication of protocols where data collection has already started, in which case the scope for the review process to influence the study design is even more limited (e.g. data collection methods might not be amenable to adjustment). This can allow more efficient workflow, but would mostly be appropriate for long-duration studies, because the smaller the proportion of data available at the time of protocol submission, the less that the analysis plan could be biased by knowledge of results.

Second, even moderately-sized clinical studies can take many months to execute, and the execution phase is almost invariably preceded by organisational and funding-related bureaucracy, as well as complex ethics review procedures, which also imply large investments of time and effort. Publishing the protocol in the interim creates a citable record that showcases ongoing work, and helps the field to determine priorities and avoid duplication of effort. It can also be professionally valuable in terms of expediting the development of a publication track record (e.g. in the case of early career researchers).

Third, the high resource implications of most clinical research often inclines researchers to design studies that maximize data yield, which may mean including multiple arms or types of measure (e.g. behavioural, physiological, anatomical). A published protocol can provide a common reference point for multiple papers that result from separate measures or sub-analyses, and can be used to mitigate against the pitfalls (in terms of research transparency) of the compressed methods that may be encouraged by high-impact publication venues and/or those targeted at time-pressed clinical audiences.

Fourth, although published protocols make the study plan public, they are not binding, so researchers retain the autonomy to deviate as required from the original plan. In this sense, published protocols are more flexible than Registered Reports. As outlined in an earlier section, flexibility is often needed to deal with the challenges of doing research in a clinical environment and with hard-to-reach samples. The down-side of this flexibility is that readers cannot trust reflexively in the level of bias control achieved by a study with a published protocol, but need to carefully examine any deviations from the plan, and the rationale for them.^19^ As for published protocols, deviations should be declared and explained in the final report, although no formal mechanisms exist to ensure this. At a minimum, deviations will be traceable by a comparison of the protocol and the final paper, and published protocols are generally more discoverable than repository preregistrations.

In summary, a published protocol requires more time and effort than repository preregistration. It usually includes a full report of the background, rationale, and methods for a study, and it undergoes a formal peer-review process. This creates more opportunities to enhance the quality and rigour of the study design, and the greater discoverability of published protocols makes the transparency more assured. It could be argued that the greater visibility of a published protocol also implies a firmer commitment to publication of the final results, regardless of outcome, potentially reducing publication bias. Nonetheless, the publication of a protocol generally implies no guarantee that the final report will be published; this is a separate process, usually at another journal, without continuity of reviewers or editorial process. Therefore, this form of preregistration does not protect entirely against (implicit or explicit) pressure to produce positive results for publication.

### Standard registered reports

The strongest form of bias control is achieved via a Registered Reports, an article format launched at *Cortex* in 2013^8,20^ and now adopted by over 300 journals across diverse disciplines.^21^ In its standard form, a defining characteristic of the format is that the journal makes a publication decision based on a peer-reviewed study plan prior to any data collection. If the editor and reviewers determine that the study can answer a coherent question of sufficient scientific interest, then *In Principle Acceptance* will be awarded. *In Principle Acceptance* of the (Stage 1) plan guarantees publication of the final (Stage 2) paper, regardless of how the results turn out, provided that the authors follow the agreed methods and add an appropriate discussion. Unlike a published protocol, the Stage 1 manuscript is not a standalone publication (although it is a citable object for the *in progress* study), but it ultimately becomes the Introduction and Methods sections of the final Stage 2 report. Standard Registered Reports also stand out from the other preregistration routes because of strict limitations imposed on flexibility in the execution of the research. The two-stage publication process forces authors to fix their hypotheses, methods, and analysis plans in advance, and subsequent changes to the protocol can only be minor, and require editorial approval. Authors are at liberty to add exploratory analyses at Stage 2, but these *post-hoc* elements cannot drive the main conclusions.

Registered Reports neutralize the most prevalent and pernicious sources of bias in the literature. First, evaluations of scientific quality are decoupled from knowledge of results, so editors and reviewers cannot prefer positive or ‘exciting’ findings, removing publication bias. Second, it guards against selective reporting. and other forms of ‘p-hacking’, whether intentional or unintentional. Third, the Introduction and Methods of the manuscript are fixed at Stage 1, so there can be no post-hoc reframing to more neatly ‘predict’ observed outcomes (i.e. HARKing).^2^ While all forms of preregistration aim to prevent p-hacking and HARKing, Registered Reports render these practices all but impossible. The effectiveness of the format is corroborated by initial meta-scientific studies. Scheel, Schijen, and Lakens^22^ sampled 71 Registered Reports and 152 standard reports in the psychological literature, and found that the (first-tested) experimental hypothesis was supported by a significant result in 44% of *Registered Reports*, as compared with an alarmingly high 96% of standard reports (see also Allen and Mehler^23^).

Registered Reports commonly require higher standards of evidence than regular research articles, for instance high statistical power. *Cortex*, where the format was first established, requires authors to show that their study plan has 0.9 power at a significance criterion of 0.02, or comparably strong Bayesian standards of evidence (BF ≥ 6), for all hypotheses. Many other journals have followed suit (see Supplementary Table 1). High power, and a correspondingly large sample size, is scientifically desirable not only because null results will be less likely to be false-negatives, but because a lower proportion of significant outcomes will be likely to be false positives.^24^ Authors of Registered Reports are often encouraged not to base power calculations on central estimates of effect size from prior research, which may be biased upward if the research was published through regular routes. Instead, they will often target a lower-bound estimate, or a smallest effect size of interest defined on theoretical or practical grounds.^25,26^ Accordingly, the sample sizes of Registered Reports are often an order of magnitude greater than in regular reports.

The stringent requirements of Registered Reports place heavy demands on scientists. This could deter engagement for a field such as neuropsychology, which has so many other challenges to contend with. For instance, the increases in sample size required to move from moderate power (e.g. 0.6 or 0.8) towards the more idealized Registered Reports levels (e.g. 0.9 or 0.95) can be prohibitive when working with specialist samples, and clinical ethical committees can reject such ‘overpowered’ designs, because of the patient burden and clinical resource implications. The potential for conflict between the scientifically-ideal design and the clinically acceptable one can create severe problems for researchers who wish to apply the Registered Reports model to clinical samples. It may not be viable to submit a Stage 1 manuscript before ethical approval is guaranteed, but if Registered Reports reviewers request changes to the design, as they often do, this will entail ethical amendments, which might not be approved. Considering the need to co-ordinate, and even serialize, two arduous processes, and to satisfy two sets of reviewers with different priorities, it is little wonder that Registered Reports involving neuropsychological samples are rare (see Fig. 1B).

The additional demands of the format for authors may be offset to some extent by the guarantee of publication conferred by *In Principle Acceptance*. The ability to accommodate reviewer comments at the design stage also means that rejection rates for Stage 1 Registered Reports may be lower than for standard reports; and rejection at Stage 2 is practically unheard of.^21^ Moreover, there is some meta-scientific evidence that Registered Reports have higher methodological quality than regular reports, perhaps because of the peer review at the design stage.^27^ Of course, the greatest motivation of all for this format is the potential to establish an unbiased evidence base on questions of scientific importance. Nonetheless, to outweigh the costs of engagement, for a field like neuropsychology, there is a need for a middle ground, which is sensitive to the challenges faced by researchers in the field. Fortunately, the standard format has now evolved into a wider family of more flexible and accessible models, as we shall outline in the following section.

### Flexible registered reports

The core benefit of Registered Reports is unbiased publication, which depends mainly on scientific evaluations being blind to results. Standard Registered Reports often also require high standards of evidence, implying large sample sizes, and impose strict constraints on the flexibility and timelines of study execution. This multiplies the barriers to engagement for neuropsychological research, and risks alienating researchers in the field from this valuable route to publication. Recently, alternative models for Registered Reports have begun to emerge that retain the goal of unbiased publication but allow degrees of flexibility that make the format accessible to more diverse research approaches.

First, there can be flexibility in required standards of evidence. Although many journals offering *Registered Reports* have high demands for power or sensitivity, this is not universal. Instead, the editor and reviewers can evaluate each Stage 1 submission on its own merits, taking into account practical constraints (including rarity of specialist samples), and make an informed judgement about whether the study is sufficiently well-designed that its outcomes will carry a worthwhile message for the field. Second, there can be flexibility in the level of bias control. As the *Registered Reports* format has gained traction, there has been a drive to accommodate secondary data analyses (including meta-analyses) and other approaches in which some prior exposure to the data may be unavoidable. In this context, it is recognized that data exposure can occur to differing degrees and that this can be transparently declared. Degrees of data exposure can be stratified into six levels of bias control, where the standard *Registered Report* defines the maximum level (Level 6), with five progressively weaker levels below it (see Table 4). As the level of bias control is reduced, the format becomes more accessible not only to secondary data studies, but also to primary studies where data collection may already have begun. After all, there is no essential difference in terms of data exposure between a secondary study for which the authors have had partial data access, and a primary study for which data collection is ongoing.

**Table 4:** Six levels of bias control, stratified by the risk of data exposure prior to acceptance of the stage 1 registered report, as defined by peer communities in registered reports

Level	Data already exist or will exist prior to IPA	Data are accessible to the authors	Data have been accessed by the authors	At least some data have already been observed by the authors	Key variables in the data have been observed by the authors	Authors have already analysed key variables	Risk of bias due to prior data observation	Multi-disciplinary inclusivity
6	*Level 6 description:* No part of the data or evidence that will be used to answer the research question yet exists and no part will be generated until after IPA (so-called ‘primary RR’)							
	**✕**	**✕**	**✕**	**✕**	**✕**	**✕**	Zero	Very low
5	*Level 5 description:* ALL of the data or evidence that will be used to answer the research question already exist but are currently inaccessible to the authors and thus unobservable prior to IPA (e.g. held by gatekeeper)							
	**✓**	**✕**	**✕**	**✕**	**✕**	**✕**	Very low	Very low
4	*Level 4 description:* At least some of the data/evidence that will be used to answer the research question already exists AND is accessible in principle to the authors (e.g. residing in a public database or with a colleague) BUT the authors certify that they have not yet accessed any part of that data/evidence							
	**✓**	**✓**	**✕**	**✕**	**✕**	**✕**	Low	Low
3	*Level 3 description:* At least some data/evidence that will be used to the answer the research question has been previously accessed by the authors (e.g. downloaded or otherwise received), but the authors certify that they have not yet observed ANY part of the data/evidence							
	**✓**	**✓**	**✓**	**✕**	**✕**	**✕**	Moderate	Moderate
2	*Level 2 description:* At least some data/evidence that will be used to answer the research question has been accessed and partially observed by the authors, but the authors certify that they have not yet sufficiently observed the key variables within the data to be able to answer the research question AND they have taken additional steps to maximize bias control and rigour (e.g. conservative statistical threshold; recruitment of a blinded analyst; robustness testing, multiverse/specification analysis, or other approach)							
	**✓**	**✓**	**✓**	**✓**	**✕**	**✕**	High—additional steps required to control bias	High
1	*Level 1 description:* At least some of the data/evidence that will be used to the answer the research question has been accessed and the authors HAVE sufficiently observed the key variables to be able to answer the research question, but the authors certify that they have not yet performed ANY of their preregistered analyses, and, in addition, they have taken stringent steps to reduce risk of bias. Such measures will be similar to the countermeasures required for Level 2 but even more intensive, including an extremely conservative statistical threshold, recruitment of a blinded analyst, comprehensive robustness testing, the use of a broad multiverse/specification analysis, or other approaches for controlling risk of bias.							
	**✓**	**✓**	**✓**	**✓**	**✓**	**✕**	Very high—stringent steps required to control bias	Very high

This table is taken from the guide for authors at https://rr.peercommunityin.org/help/guide_for_authors. The highest level of bias control (Level 6) corresponds to the lowest risk of data exposure, which is the standard model for a Registered Report based on primary data, in which no data have been collected prior to Stage 1 acceptance. Lower levels of bias control allow more risk of data exposure, making the format accessible to a wider range of research approaches. Levels 2–5 are common scenarios for Registered Reports based on the secondary analysis of existing data. These levels can also make it possible to submit a Stage 1 Registered Report for a primary study in which data collection is underway but incomplete. It could be argued that only studies at the top levels of bias control (Levels 6 and 5) should carry the name Registered Reports, because they are the only cases in which we can be certain that the study protocol has been fixed prior to any knowledge of results. We leave this definitional issue to one side. The practical point is that flexibility of bias control can alleviate restrictions on the study time course, whilst still providing In Principle Acceptance based on a results-blind assessment of scientific quality.

Flexibility of level of bias control relaxes the pressure on study timelines in neuropsychology, because it removes the need to serialize the Stage 1 review process and data collection. Instead, it becomes possible to start collecting data immediately upon ethical approval, and to submit a Stage 1 manuscript in parallel. Unless data collection progresses very rapidly, it should be possible to fix the level of bias control at Level 3, or if direct access to the data can be avoided until collection is complete, then bias control can stay at Level 4 or even Level 5. Of course, if the study is already underway then the Stage 1 review process may not be able to influence the design of data collection, and so the benefit of reviewer input into study design is lost, although reviewers may still be able to fine-tune the analysis plan. The Stage 1 review process will thus be more like that for a standard research article, in which the logic and design of an existing study are evaluated. Crucially, however, even at the weakest levels of bias control (Level 2 or 1), the editorial evaluation of scientific quality will still be made blind to results, preserving this key feature of the Registered Reports framework.

These flexible models have yet to be explicitly adopted by journals, but they are implied in the author guidelines of some outlets. To help steer colleagues in this respect, we summarize in Supplementary Table 1, the requirements of journals that offer Registered Reports, and at which neuropsychology research would be within scope. There is variation in the required standard of evidence, with some outlets having no explicitly defined minimum (this implies consideration on a case-by-case basis, but any ambiguity in the guidelines should be checked with the journal office). It is rare for the minimum level of bias control to be explicitly stated, but the guidelines can usually be mapped quite easily to the bias control levels in Table 4 (we provide further notes on this in Supplementary Table 1). Where journal guidelines explicitly distinguish between primary and secondary data approaches, the required level of bias control is usually Level 6 for primary research studies (i.e. with novel data collection), with a reduced level permitted for secondary analyses.

However, full flexibility for primary or secondary data is explicitly the default policy at *Peer Community In Registered Reports* (PCI RR; rr.peercommunityin.org). PCI RR is not included in Table 4, because it is not a journal: it is a journal-independent, non-profit platform for the review and editorial recommendation of Stage 1 and Stage 2 Registered Reports across the sciences. Researchers can submit their Stage 1 manuscript as a (public or private) preprint to PCI RR, declaring their standard of evidence and level of bias control. They have the option to nominate ‘Recommenders’, analogous to an action editor, who will evaluate the manuscript, and may send it for peer review. Informed by reviewers’ advice, the Recommender can effectively award *In Principle Acceptance* by ‘recommending’ a Stage 1 study plan. Following the completion of the study, the subsequent Stage 2 manuscript is also submitted to PCI RR, who will publish a formal recommendation of the final manuscript (with an open review history), provided that the authors have followed the agreed protocol and added an appropriate discussion.

The PCI RR process confers the same validation as journal-based academic peer-review, and the open-access preprint becomes a citable peer-reviewed output. Should the authors wish to place their paper in a traditional journal thereafter, they have options available in the form of ‘PCI RR-interested’ and ‘PCI RR-friendly’ journals. PCI RR-interested journals are partner journals that receive alerts about recommendations within their scope. They may contact the authors to offer publication, or authors can approach the journal for consideration. Acceptance is not automatic, and a PCI RR-interested journal may perform further evaluations and request revisions. A stronger commitment is made by PCI RR-friendly journals, who will accept—*without further peer review*—a recommended Stage 2 manuscript, provided that it meets their disciplinary scope and criteria for minimum standards of evidence and level of bias control. Note that these criteria are sometimes more lenient for papers received via the PCI RR route than they are papers submitted direct to the journal; for instance, *Brain & Neuroscience Advances, Cortex,* and *Royal Society Open Research* all currently have Level 2 as the minimum level of bias control for any submission via the PCI RR route. This allows for Stage 1 submission to PCI RR of primary data studies that are underway but incomplete.

## Further considerations for neuropsychologists

When we surveyed research-active UK neuropsychologists about their engagement with open research, their responses highlighted some quite specific concerns about preregistration, as well as some possible misunderstandings about the relation to other open research practices. We thus felt it wise to include an additional section below, to address two of the main issues raised.

### Single-case designs

Single-case studies have played a key role in the history of neuropsychology, and remain an important method for basic and translational research^28-30^. They are not, however, perceived by many as having a good fit with preregistration. The archetypal case study is an in-depth exploration of patterns of impairment across a range of cognitive and behavioural abilities in a person with brain damage. These studies are often opportunistic and time pressured, because the window for testing the person might elapse, and they can be inherently reactive, with tests designed ‘on the fly’ to probe unexpected patterns. These traditional characteristics of single-case studies are in tension with the requirements of preregistered research: at least some of the data have already been seen (that is how the person became of interest), and the study plan depends reactively on observations made (it cannot be nailed down in advance). If the statistical approach depends on comparing single cases against normative control samples, then there are hard limits on achievable power^31^; and even if the approach is based on between-condition comparisons within a single-case, the number of observations may be limited by the person’s ability to concentrate for long periods, or the testing time available in clinical settings.

This general class of method may therefore seem to be antithetical to preregistration. However, there are several scenarios in which case study approaches could be preregistered to good advantage. First, some neuropsychological conditions are relatively stable, and some people with neuropsychological conditions are exemplary experimental participants. Indeed, there are well-known cases that have participated in research studies over years and even decades^32-35^. Such stable cases present the possibility of prospectively generating and preregistering novel predictions about an individual’s abilities, which can be tested on a planned schedule. Second, single-case studies can be concatenated into case-series, which estimate how common certain patterns of individual impairment are within a population defined on some independent criterion, such as neuropsychological diagnosis or location of brain damage. In such projects, where the sample is identified a priori, it should be possible to create a preregistered protocol, and even to submit a registered report, to specify how testing and analysis will proceed. Indeed, one of the few *Registered Reports* so far conducted with neuropsychological samples is an extended case-series replication of an influential single-case study of neglect, albeit this study did encounter significant challenges in execution.^36^

Finally, an important class of single-case study for applied work is Single Case Experimental Design (SCED), also known as ‘N-of-1 trials’.^28,37,38^ SCEDs use longitudinal measurement to chart an individual’s response to an intervention on some target behaviour, typically through phases of baseline, treatment, and withdrawal of treatment. This can provide important preliminary evidence for treatment efficacy where group studies are not feasible, perhaps because the condition studied is rare. These studies fall under the definition of clinical trials and should accordingly be required to publish the protocol in advance. There is no trial registry dedicated to SCEDs, but there is value in preregistering via clinical trial registries (such as on clinicaltrials.gov), or other available routes.

### Ethical sharing of neuropsychological data

Funders, regulatory bodies, professional societies, and journals are increasingly requiring some form of data sharing. Even if one is not compelled to share, then arguably we are morally obliged to return the data to the public domain from whence it was collected and probably paid for. Legal and ethical reasons aside, there are at least two key scientific benefits: first, it enhances transparency and reproducibility, and invites more robust scrutiny of claims; second, it facilitates collaboration and discovery by allowing researchers to revisit or combine datasets in attempts to replicate or extend findings. For these reasons, researchers should incorporate data retention and sharing clauses into ethics templates, as far as is possible. However, they must also be mindful of potential risks, particularly when the data contains sensitive (e.g. medical) and identifiable information. Neuropsychological data on an individual may be especially sensitive given it can be used to inform decisions about someone’s capacity to consent to treatment, and regarding their ability to drive or to care for themselves or others, for example. Thus, careful consideration needs to be given to the level of data sharing, with particular attention to protecting participant identities.

The results of our survey suggest it is presumed among some neuropsychologists that to preregister research, there is a requirement to commit to ‘open data’. It is not, in fact, an explicit requirement, even for the strongest forms of preregistration. In practice, though, the extent to which data can be shared may influence the route to preregistration that is chosen for a study. This is because restrictions on access may limit the ability of reviewers of Stage 2 Registered Reports to scrutinize the degree to which study plans were adhered to. There may be a tension between the need to share as much of the data as possible, to ensure transparency and facilitate discovery, and the need to restrict access to protect privacy. Fortunately, there is no singular way in which data sharing must be undertaken, and there are various means by which the accessibility, and thereby the value of datasets, can be maximized while avoiding added risk to participants. We provide examples below.

When sharing sensitive data, like neuropsychological test results, implementing robust de-identification procedures is key. Common practices include removing non-essential demographic information, and sharing only processed or summary data (e.g. test scores) rather than raw data (which could be identifiable handwriting samples or voice recordings, for example). Where this would compromise the quality or value of the dataset, then it is possible to share the finer-grained data but restrict who can access it and how. For example, there are different platforms for data sharing, from public repositories that are open to the public (e.g. the OSF), to those that restrict access to only qualified researchers who have signed a data-use agreement. Some large cohort databases (e.g. UK BioBank; Dementias Platform UK) are now accessed via remote analysis platforms, which allow approved researchers to perform secondary analyses, while the data remain on secure servers. Many practical tips for effective and ethical sharing of data from human participants are proffered in a thoughtful tutorial by Meyer.^10^ While data sharing is not necessary for preregistration, it is scientifically desirable in its own right and, together, these practices maximize the value of research.

## Summary and recommendations

Preregistration is a tool to encourage careful planning, improve transparency of research process, and enhance the rigour of one’s science. Opening up study plans to external scrutiny before data collection, and publishing the plans themselves, are additional steps that help maximize the quality of research, and empower readers to make informed assessments of reliability. To the extent that preregistration can limit undisclosed flexibility in study execution, it limits likely sources of bias; and the Registered Reports format—the strongest form of preregistration—can in principle ensure unbiased evidence. We believe it desirable for the health of neuropsychology, and cognate disciplines, that we continue the trend towards adoption of preregistration practices, but this requires us to acknowledge and address the real and perceived barriers presently limiting uptake.

There are numerous non-trivial challenges increasing the difficulty of preregistering neuropsychological research, particularly via standard Registered Reports. However, new and more flexible models are emerging that make preregistration more accessible to researchers facing such challenges. We have tried to emphasize that preregistration is not an all-or-none proposition: there are different forms of preregistration. These lie roughly along a continuum in which rigour and bias control are traded off against flexibility of execution and timing. We have mapped out key issues and decision points, while signposting to resources and outlets, with the aim of empowering colleagues to choose forms of preregistration that work best for their research objectives and practical constraints. Preregistration is not appropriate for all research stages or questions, and nor can it guarantee high-quality science, but it is a powerful practice for hypothesis-testing research, and transparency of process is *always* better than opacity. If neuropsychologists can embrace the logic and practices of preregistration, not only can we improve the quality of evidence in our field, but we can also play our part in shaping and advancing open research more broadly.

## Supplementary Material

fcaf162_Supplementary_Data

## Data Availability

Original data for this report is publicly available, along with R code to reproduce the visualisations of data in Fig. 1, at https://osf.io/n7kdv.
