# Supplementary material for: Practical routes to preregistration: a guide to enhanced transparency and rigour in neuropsychological research
Source: Brain Commun. 2025 Apr 28;7(3):fcaf162. doi: 10.1093/braincomms/fcaf162 (PMC12066951; doi:10.1093/braincomms/fcaf162)
Supplement: fcaf162_Supplementary_Data [file fcaf162_supplementary_data.docx]

**Supplementary Materials**

for

**Practical Routes to Preregistration: A Guide to Enhanced Transparency and Rigour in Neuropsychological Research**

Richard J. Binney, Laura J. Smith, Stephanie Rossit, Nele Demeyere, Gemma Learmonth, Elena Olgiati1, Ajay D. Halai, Elisabeth Rounis, Jonathan Evans, Nicola M.J. Edelstyn & Robert D. McIntosh

**Supplementary Table 1. Journals advertising a Registered Reports article type for which neuropsychology research would be within scope (including some quite general outlets).**

| **Journal/Platform** | **Publisher** | **Minimum standard of evidence** | **Minimum bias control level** |
| --- | --- | --- | --- |
| *Behavioral Neuroscience* | American Psychological Association | Not stated | Unclear, but suggests that submission must precede study (≡ L6) |
| *Brain & Behavior* | Wiley | Not stated | Unclear, but suggests that submission must precede study (≡ L6) |
| *Brain & Cognition* | Elsevier | power .90; α .05*  1/6 < BF > 6 | Unclear: pre-submission enquiry recommended if authors are in possession of the data, or have observed any part of it. |
| *Brain & Neuroscience Advances* | Sage | power .80; α variable  1/6 < BF > 6 | 1ry RR: before data collection (≡ L6)  2ry RR: before critical analyses (≡ L2) |
| *Brain Communications* | Oxford University Press | Not stated | Stage 1 submission before carrying out experiments  (≡ L6) |
| *Cogent Psychology* | Taylor & Francis | Power .90; α .05*  1/6 < BF > 6 | 1ry RR: before data collection (≡ L6)  2ry RR: before data access (≡ L4) |
| *Cortex* | Elsevier | power .90; α .02  1/6 < BF > 6 | 1ry RR: before data collection (≡ L6)  2ry RR: before critical analyses (≡ L2) |
| *Developmental Cognitive Neuroscience* | Elsevier | Not stated | 1ry RR: before data collection (≡ L6)  2ry RR: before critical analyses (≡ L2) |
| *European Journal of Neuroscience* | Wiley | power .90; α .05  1/6 < BF > 6 | 1ry RR: before data collection (≡ L6)  2ry RR: before data access (≡ L4)  Presubmission enquiries for other cases. |
| *Journal of Cognitive Neuroscience* | MIT press | Not stated | Unclear, but suggests that submission must precede study (≡ L6) |
| *Journal of Neuropsychology* | British Psychological Society | power .90; α .05*  BF not stated | Unclear, but suggests that submission must precede study (≡ L6) |
| *Laterality: Asymmetries of Body, Brain, and Cognition* | Taylor & Francis | power .90; α .05*  1/6 < BF > 6 | 1ry RR: before data collection (≡ L6)  2ry RR: before data access (≡ L4) |
| *Nature Human Behavior* | Nature | power .95; α .05*  1/10 < BF > 10 | 1ry RR: before data collection (≡ L6)  2ry RR: before data access (≡ L4) |
| *Neuroscience of Consciousness* | Oxford Academic | Power not stated, α not stated  1/3 < BF > 3 | 1ry RR: before data collection (≡ L6)  2ry RR: before data access (≡ L4) |
| *PeerJ* | PeerJ | No minimum | Level 1 |
| *Plos ONE* | PLOS | Not stated | Not stated |
| *Psychological Science* | Sage | Not stated | 1ry RR: before data collection (≡ L6)  2ry RR: before data access (≡ L4)  Presubmission enquiries for other cases. |
| *Royal Society Open research* | Royal Society Publishing | Not stated | 1ry RR: before data collection (≡ L6)  2ry RR: before data access (≡ L4) |
| *Scientific Reports* | Nature | Not stated | 1ry RR: before data collection (≡ L6)  2ry RR: before data access (≡ L4) |

*Note:* *The criteria listed in columns 3 and 4 reflect our interpretation of the author guidelines provided by the journal. For minimum standard of evidence, an asterisk is appended in cases in which the alpha (α) level is not specified, and we have assumed .05 as the default level. For minimum level of bias control, journal guidelines rarely refer to a numerical level of bias control, so the numerical level is our interpretation of the equivalent level from Table 5 (e.g. ≡ L6 means that the criteria imply a minimum Level 6). The bias control criteria usually differ for primary (1ry) studies, which we take to imply novel data collection, and secondary (2ry) studies, which we take to imply analyses of pre-existing data. A longer list of journals offering Registered Reports across all fields, with links to author guidelines, is at* [*https://www.cos.io/initiatives/registered-reports*](https://www.cos.io/initiatives/registered-reports)*.*
